# Socioeconomic status and infectious intestinal disease in the community: a longitudinal study (IID2 study)

**DOI:** 10.1093/eurpub/ckx091

**Published:** 2017-08-02

**Authors:** Natalie L Adams, Tanith C Rose, Jeremy Hawker, Mara Violato, Sarah J O’Brien, Margaret Whitehead, Benjamin Barr, David C Taylor-Robinson

**Affiliations:** 1 NIHR Health Protection Research Unit in Gastrointestinal Infections, Liverpool, UK; 2 Department of Public Health and Policy, University of Liverpool, Liverpool, UK; 3 National Infection Service, Public Health England, London/Birmingham, UK; 4 Health Economics Research Centre, University of Oxford, Oxford, UK

## Abstract

**Background:**

Infectious intestinal diseases (IID) are common, affecting around 25% of people in UK each year at an estimated annual cost to the economy, individuals and the NHS of £1.5 billion. While there is evidence of higher IID hospital admissions in more disadvantaged groups, the association between socioeconomic status (SES) and risk of IID remains unclear. This study aims to investigate the relationship between SES and IID in a large community cohort.

**Methods:**

Longitudinal analysis of a prospective community cohort in the UK following 6836 participants of all ages was undertaken. Hazard ratios for IID by SES were estimated using Cox proportional hazard, adjusting for follow-up time and potential confounding factors.

**Results:**

In the fully adjusted analysis, hazard ratio of IID was significantly lower among routine/manual occupations compared with managerial/professional occupations (HR 0.74, 95% CI 0.61–0.90).

**Conclusion:**

In this large community cohort, lower SES was associated with lower IID risk. This may be partially explained by the low response rate which varied by SES. However, it may be related to differences in exposure or recognition of IID symptoms by SES. Higher hospital admissions associated with lower SES observed in some studies could relate to more severe consequences, rather than increased infection risk.

## Introduction

Infectious intestinal disease (IID) is common, leading to diarrhoea, vomiting and, occasionally, more serious complications such as renal failure. Previous estimates suggest around 25% of people in UK suffer an episode of IID per year^1^ and that foodborne illness in England and Wales costs individuals, the economy and NHS around £1.5 billion annually.^2^ Many infections are socially patterned, however, the role of socioeconomic status (SES) in risk of IID in developed countries, such as UK, is not well understood.^3^

A large proportion of the burden of IID remains hidden; it is estimated that there are 147 cases in the community for every one case reported to national surveillance;^2^ many individuals do not present to healthcare as most infections are self-limiting. Additionally, it is unclear whether socioeconomic patterns reported in hospital and laboratory-based surveillance reflect differences in risk of infection or in reporting and healthcare-seeking behaviour.^4^ Longitudinal population-based survey data can provide better estimates of differences in risk of infection that may not be captured through routine surveillance. This study aims to explore whether different socioeconomic groups experience different risk of IID in the UK, through the analysis of a large prospective population cohort, to improve understanding of the role of SES in IID in the community and to inform policies to reduce health inequalities. In this study, we provide an up-to-date assessment of the association between IID and SES for all ages in UK.

## Methods

### Design, setting and data source

We undertook a longitudinal analysis of data collected through a large prospective community cohort in UK (IID2 study).^1^^,^^2^ A cohort of 6836 randomly selected participants was recruited from 88 representative general practices in UK. Sociodemographic information including age, gender and occupation were obtained through a baseline survey upon entry to the cohort and details of IID symptoms were recorded on a weekly basis for up to 1 year, from October 2007 to August 2009, through the return of an email or postcard indicating whether symptoms of diarrhoea and/or vomiting had been experienced in the previous week. Individuals who reported symptoms completed a more in-depth questionnaire through which details of illness and healthcare contact were recorded.

Overall participation rate was low (9%) and individuals who declined to participate were younger, more deprived, living in urban rather than rural areas and employed in lower supervisory and technical occupations.^2^ The 6836 participants contributed 4658 person-years of follow-up; median follow-up duration was 39 weeks.^2^ Among participants, no differences in follow-up were identified by sex, SES or rural–urban classification.^2^ Average follow-up time was similar for those who experienced an episode of IID and those who did not.^2^ Managerial/professional occupations were over-represented in the study, while intermediate, and semi-routine and routine occupations were under-represented, in comparison to the UK population.^2^ Those of White ethnicity were slightly over-represented.^2^

Ethical approval and informed consent were originally obtained for the main study (07/MRE08/5). This included the provision to use the data for future research. Approval for this secondary analysis of the fully anonymised datasets was not required.^2^

Infectious intestinal disease was defined as loose stools or clinically significant vomiting (vomiting occurring more than once in 24 h and if it incapacitated the case or was accompanied by other symptoms such as cramps or fever)^2^ lasting <2 weeks, in the absence of a known non-infectious cause, preceded by a symptom-free period of 3 weeks.^2^ Cases experiencing illness considered to be travel-related were excluded.

The primary exposure of interest was an individual-level measure of SES, self-reported occupation, with each individual assigned a National-Statistics Socioeconomic Classification (NS-SEC) using the five-class self-coded version.^5^ For participants aged less than 16 years, NS-SEC was assigned based on the occupation of the head of the household. For the purposes of this study, the NS-SEC variable was recoded into the three-class version to provide a hierarchy of SES, with routine/manual occupations assumed equivalent to low SES and managerial/professional occupations to high SES.^5^

### Analysis strategy

Analyses were conducted in Stata 13.1 (Statacorp, TX). Rates of IID within the study population and by SES were calculated accounting for follow-up time, to produce rates of IID per 1000 person-years with associated 95% confidence intervals. The main analysis investigated the relationship between SES, as measured by NS-SEC, and time to first IID episode for each participant using Cox proportional hazard regression modelling, with subsequent episodes of IID for an individual being dropped. We first explored univariate relationships between SES and the covariates of interest [rurality and employment status (employed/not working)] before fitting a multivariate Cox proportional hazard regression model, adjusting for the potentially confounding covariates and stratifying the baseline hazard on age and sex. Kaplan–Meier survival curves were estimated to check the proportional hazards assumption. Interaction terms between the socioeconomic variable NS-SEC and each variable in turn were tested for inclusion to investigate whether the strength of any relationship was moderated by the inclusion of another variable.

We undertook a number of robustness tests, firstly allowing individuals with multiple episodes of IID to re-enter the cohort following a period of censoring (due to symptoms meeting the case definition and requiring a censored period of 3 weeks after cessation of symptoms; non-response; or symptoms not meeting the case definition), accounting for clustering within individuals by using a robust estimate of variance allowing for inter-person correlation.

We repeated the analysis using a less sensitive case definition, whereby individuals reporting symptoms which could not be verified against the case definition (due to a lack of further details about foreign travel or symptom duration) were also included as cases in the analysis. We repeated the analysis including those unclassifiable within NS-SEC to investigate whether this had an impact on the results. This NS-SEC group comprised individuals for whom it was not possible to classify their occupation or who did not respond to occupation questions.

Stratification by age group was conducted to determine whether there were differences in the rate of IID by SES for children, adults and older participants. We repeated the analysis using an area-level measure of SES, the Index of Multiple Deprivation (IMD),^6^ assigned to each individual based on their postcode.

As there were missing NS-SEC data for a group of participants for whom it was not possible to classify their occupation or who did not respond to the occupation question, Multiple Imputation using chained equations (MICE)^7^ was used in order to include these cases.

## Results

### Characteristics of participants

Of the 6836 participants in the cohort, 998 individuals reported an episode of IID during 4583.5 person-years of follow-up. Fifty-two percent (*n* = 3557) were from managerial/professional occupations, 15% (*n* = 1002) were from intermediate occupations and 17% (*n* = 1165) were from routine/manual occupations, compared with 31%, 22% and 33% respectively in the general population.^8^ For 1112 individuals (16.3%), NS-SEC was missing either because they did not respond to occupation questions or, if they did, it was not possible to classify their occupation according to the NS-SEC categories. SES was associated with age group, sex, rurality, employment status and the method of follow-up that participants elected to use (email or postcard). It was independent of ethnicity (table 1). Mean follow-up time was similar between NS-SEC groups.

**Table 1:** Characteristics of cohort participants (*n* = 6836)

	Managerial/professional *n* (%)	Intermediate *n* (%)	Routine/manual *n* (%)	Not classifiable *n* (%)	*P* value
**Total**	3557 (52.0)	1002 (14.7)	1165 (17.0)	1112 (16.3)	
**Incidence rate/1000 PYs**	235.4	243.9	166.3	194.0	
**Follow-up time** (mean days)	242.1	240.6	245.2	257.4	
**Age** (mean)	47.2	48.5	49.3	53.0	
**Case**					
Yes	555 (55.6)	161 (16.1)	130 (13.0)	152 (15.2)	0.001
No	3002 (51.4)	841 (14.4)	1035 (17.7)	960 (16.4)	
**Age group**					
<18	605 (55.5)	152 (13.9)	178 (16.3)	156 (14.3)	<0.001
18–64	2095 (54.6)	593 (15.4)	627 (16.3)	525 (13.7)	
65+	857 (45.0)	257 (13.5)	360 (18.9)	431 (22.6)	
**Sex**					
Female	2175 (52.3)	669 (16.1)	640 (15.4)	676 (16.3)	<0.001
Male	1382 (51.6)	333 (12.4)	525 (19.6)	436 (16.3)	
**Ethnicity**					
White	3464 (52.0)	981 (14.7)	1145 (17.2)	1077 (16.2)	0.125
Non-White	93 (55.0)	21 (12.4)	20 (11.8)	35 (20.7)	
**Rurality**					
Urban	2522 (50.8)	694 (14.0)	958 (19.3)	789 (15.9)	<0.001
Rural	1034 (66.8)	307 (19.8)	206 (13.3)	323 (17.3)	
**Follow-up**					
Email	2564 (60.3)	622 (14.6)	529 (12.4)	539 (12.7)	<0.001
Postcard	993 (38.5)	380 (14.7)	636 (24.6)	573 (22.2)	
**Employment status**					
Employed	2493 (56.7)	713 (16.2)	769 (17.5)	423 (9.6)	<0.001
Not working	1061 (44.1)	287 (11.9)	396 (1.5)	664 (27.6)	

Notes: PYs, person-years. Missing data: Employment status was missing for 30 individuals. Rural–urban classification was missing for three individuals.

**Figure 1:**
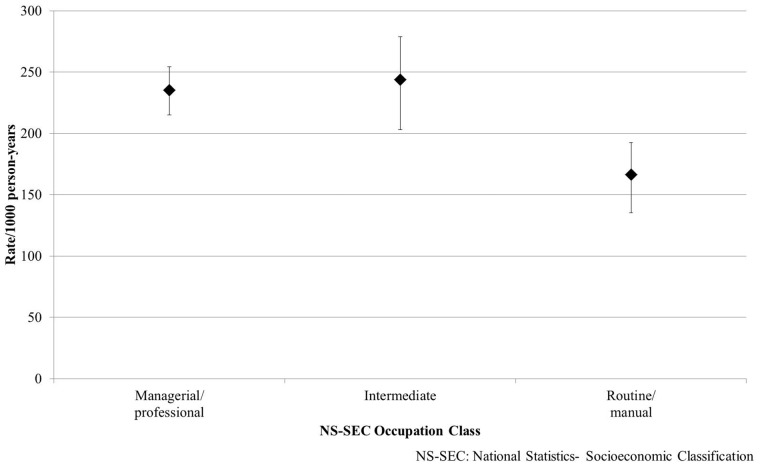
Incidence rates per 1000 person-years by NS-SEC classification. NS-SEC, National Statistics-Socioeconomic Classification

Incidence was lower among routine/manual occupations compared with managerial/professional occupations (166.3/1000 person-years, 95% CI 140–197; 235.4/1000 person-years, 95% CI 217–256 (figure 1). 

### Main analysis

Participants for whom NS-SEC was not classifiable were excluded from the main analysis; 5724 participants were included. All potentially confounding variables were retained in the fully adjusted model; ethnicity and follow-up type were excluded as these were not considered to be confounders (table 2). IID hazard was significantly lower in routine/manual occupations compared with managerial/professional occupations (HR 0.74, 95% CI 0.61–0.90). No significant interactions were identified.

**Table 2:** Adjusted and unadjusted Cox regression analysis (*n* subjects = 5716; *n* failures = 845)

Variable	Category	Unadjusted		Adjusted^a^		*P* value
		Hazard ratio	(95% CI)	Hazard ratio	(95% CI)	
**NS-SEC**	Managerial/professional	1.0 (reference)		1.0 (reference)		
	Intermediate	1.04	(8.87–1.23)	1.03	(0.86–1.23)	0.74
	Routine/manual	0.71	(0.58–0.86)	**0.74**	**(0.61–0.90)**	**0.002**
**Rurality**	Urban	1.0 (reference)		1.0 (reference)		
	Rural	1.17	(1.01–1.36)	1.13	(0.98–1.31)	0.09
**Employment status**	Employed	1.0 (reference)		1.0 (reference)		
	Not working	0.78	(0.67–0.91)	1.00	(0.82–1.22)	1.00

Notes: Baseline hazard stratified by age group and sex. Missing data: NS-SEC was not classifiable for 1112 individuals. Employment status was missing for five individuals. Rural–urban classification was missing for three individuals. NS-SEC, National Statistics-Socioeconomic Classification; CI: confidence interval.

a Adjusted for all other covariates in the model.

### Sensitivity analyses

The lower hazard in routine/manual occupations compared with managerial/professional occupations was a consistent finding across the sensitivity analyses accounting for multiple spells of follow-up; using a less sensitive case definition; including the not-classifiable NS-SEC group; and using multiple imputation for NS-SEC (Appendices B–E).

In the models stratifying for age (Appendices F.1–F.3), the Hazard Ratio for routine/manual occupations compared with managerial/professional occupations tended to decrease with increasing age (65 and over: 0.60, 95% CI 0.40–0.89, *P* = 0.012; 0–17 years: 0.89 (95% CI 0.61–1.29, *P* = 0.54), however, these differences were non-significant.

Using the area-level IMD as a measure of SES, the most deprived (IMD quintile 1) had lower incidence compared with the least deprived (IMD quintile 5) (171.9/1000 person-years, 95% CI 132.6–222.8; 234.1, 95% CI 206.9–264.8) in accordance with the main analysis results. However, no statistically significant relationship was identified in the adjusted analysis (Supplementary Appendix G). The distribution of SES by IMD differed compared with the general population, with those in the most deprived quintile being underrepresented (7% versus 20%) and in the least deprived quintile (24% versus 20%) compared with the distribution in the general population.^2^ No significant interactions were identified in any of the sensitivity analyses.

## Discussion

In this analysis of a large representative UK sample following a prospective community cohort to monitor the development of IID symptoms, we investigated the relationship between IID and SES using occupation as an individual-level measure of SES. Lower SES was associated with significantly lower risk of IID. There were no significant age-stratified differences in the relationship between IID and SES.

We undertook a novel analysis of an existing population-based community cohort assessing the association of both individual and area-based measures of SES with IID. Survival analysis explored the relationship between IID and SES accounting for censored observations and different time to event for participants. Multiple sensitivity analyses were conducted to assess the robustness of the main results. A key strength of this study is that it does not require an individual to seek care or have a specimen taken in order to be included in the study, thus reducing potential bias if healthcare-seeking behaviour differs by SES.

However, participation in the cohort study was low; only around 9% of the original number recruited and screened for participation, lower than the first IID study (35%),^2^ and this varied by SES. Participation bias within cohort studies, particularly by SES, is a recognised limitation. The characteristics of the cohort population differed from the UK population, as those who were most disadvantaged were underrepresented compared with the UK population, while those who were advantaged were over-represented,^8^ and a large number of participants (*n* = 1112) could not be classified by NS-SEC. It is possible that those who agreed to participate had a different risk of IID compared with those who refused which may limit the generalisability of results. The lack of a significant difference in risk by SES for children could be related to small numbers in the stratified groups which means the study may lack power for detecting a difference, although the trend was of a lower risk for lower SES participants.

However, despite these limitations, this study represents an important analysis of a large prospective community cohort in UK which suggests differences in risk of IID by SES among the population within this study. To the best of our knowledge, this is the most comprehensive analysis of IID by SES conducted in UK. Our study differs from two earlier analyses of the IID2 data. Tam et al.^9^ used data from the IID2 study and found no significant difference in risk of multiple-spells of IID in disadvantaged compared with advantaged individuals, while Tam et al.^1^ found no significant difference in incidence by socioeconomic groups. The different findings between these papers could relate to differences in research questions which were answered using different and question-specific methods, as well as differences in the outcome; as our outcome was time to event, our paper used survival analysis to account for differing follow-up times.

Despite potential issues with participation bias by SES, cohort studies are a robust method of assessing individual-level exposures. However, few population-based cohort studies have been conducted in developed countries to investigate differences in IID risk by SES; studies investigating this relationship between age groups are particularly limited.

In a Dutch cohort study, individuals with a low level of education had significantly lower odds of gastroenteritis compared with those with a high level of education (OR 0.65, 95% CI 0.56–0.75);^10^ comparable with our adjusted estimate. Another cohort study,^11^ in Denmark, which looked at specific bacterial pathogens as opposed to IID, found an increased risk in adults in higher SES groups for most pathogens (*Campylobacter, Salmonella* Enteritidis and *Shigella*), however, the pattern was less clear in children, with no association between risk and SES for most pathogens; these findings also concur with our results.

By contrast, a Canadian cohort study^12^ found that individuals in neighbourhoods with low and medium household incomes had higher rates of IID compared with those living in neighbourhoods with high household incomes. In contrast to the other cohort studies above, including our study, the authors used physician visits rather than self-reported symptoms to define IID; when hospitalisation was used to define IID, the authors found no significant difference in rates by SES. Further, this study was designed to assess the association between environmental factors and IID incidence rather than SES specifically.

Several cohort studies which have focussed on children have found higher risk in more disadvantaged groups,^13–16^ in contrast with our findings. However, two of these studies^13^^,^^16^ were from the same survey, although used different SES measures to investigate the relationship, and specifically sampled very young children. Studies assessing SES specifically in children may be better powered or designed to investigate this relationship than studies looking at all ages, particularly as SES is more transient in children.

Many studies assessing the relationship between IID and SES in developed countries have used study designs other than population-based cohorts, such as cross-sectional population surveys, which have produced conflicting results. Some support our finding that lower SES is associated with lower risk of IID.^17–22^ These studies looked at adults specifically or all ages combined and used mainly education as a measure of SES, with the exception of one study which used occupation.^17^ Most cross-sectional population surveys, however, found no significant association,^19^^,^^20^^,^^22–31^ including three studies which found significant associations with education but not with income and occupation,^19^^,^^20^^,^^22^ suggesting that the association may vary with different measures of SES. The variability in these results also suggests that cross-sectional study designs may not provide the most robust estimates of the relationship between SES and IID.

There are several possible explanations for the finding of lower IID rates among individuals of lower SES. It may be artefactual and related to low response rate. The over-representation of advantaged individuals, or differential reporting by SES, may have resulted in a biased population. However, the sample was large, and the internal associations, which were the targets of inference within the sample population, are likely to be valid. Conversely, differences in the recognition or reporting of symptoms by SES or by healthcare seeking behaviour may partially explain the results. The results may also represent a real lower risk of IID among those who are disadvantaged through differences in exposures by SES (such as the consumption of less risky foods, reduced opportunity to eat meals outside of the home, reduced exposure to animal attractions, such as open farms, and reduced levels of foreign travel among those of a lower SES).^17^^,^^21^

There is some evidence from our study and others to suggest the existence of a relationship between IID and SES, with lower SES associated with lower rates of IID. Evidence from the literature, however, suggests that the consequences of IID are more severe for more disadvantaged population groups, with higher hospital admission rates for those of lower SES,^32–34^ and that disadvantaged children may be at higher risk of IID infections.^13–16^ Our results may underestimate the risk in disadvantaged groups and in children. While more disadvantaged individuals may be at a lower risk of, or vulnerability to, GI infections, the possibility of more severe consequences among these groups has implications for the clinical management of IID and for healthcare utilisation.

Further research is required to explore the role of symptom recognition, perception, healthcare seeking behaviour and other potentially mediating exposures to complement these results and help to explain the relationship between SES and GI infection. Focussing on children may clarify the inconsistent results seen across the literature, as would further research on the most appropriate SES measure to use to produce the most robust estimates of the association between IID and SES. Finally, a greater understanding of the individual behaviours and environmental risk factors by SES is crucial to understanding differential risk, vulnerability and consequences of IID. These results contribute to the evidence on community-level risk of GI infections. Alongside future planned analyses, this could ultimately be used to provide evidence to inform policies to address inequalities in risk, vulnerability and consequences of IID.

## Supplementary Material

Supplementary Data

## Disclaimer

The views expressed are those of the author(s) and not necessarily those of the NHS, the NIHR, the Department of Health or Public Health England.

Key points

- Gastrointestinal infections are common. There is evidence to suggest that some consequences of gastrointestinal infections, such as hospital admission, are greater in more disadvantaged individuals.
- However, the role of SES in risk of gastrointestinal infections is not well understood and studies that have investigated this relationship have presented conflicting findings.
- There is some evidence to suggest that, within the community, disadvantaged individuals are at lower risk of gastrointestinal infections compared with more advantaged individuals which could be a result of differences in exposures, healthcare seeking behaviours or symptom recognition.
