# Supplementary material for: Socioeconomic status and infectious intestinal disease in the community: a longitudinal study (IID2 study)
Source: Eur J Public Health. 2017 Aug 2;28(1):134–8. doi: 10.1093/eurpub/ckx091 (PMC5965370; doi:10.1093/eurpub/ckx091)

**SUPPLEMENTARY DATA**

The IID hazard was significantly lower in routine/manual occupations compared to professional/managerial occupations for the analysis including multiple spells of follow-up (HR 0.74, 95%CI 0.61-0.90) ,possible cases(HR 0.76, 95%CI 0.64-0.89) , the not-classifiable participants by NS-SEC (HR 0.76, 95%CI 0.65-0.89); multiple imputation to assign NS-SEC categories for those who were non-classifiable (HR 0.78, 95%CI 0.64-0.94) and using 10-year age groupings (HR 0.73, 95%CI 0.60-0.89) as for the main analysis. There was no significant relationship identified using IMD.

In the adjusted age-stratified model, there was a non-significant lower hazard in routine/manual occupations compared to professional/managerial occupations for participants aged under 18 years (HR 0.89, 95%CI 0.61-1.29); among participants aged 18-64 years and 65 and over, those with routine/manual occupations had significantly lower rates of IID compared to professional/managerial occupations (HR 0.76, 95%CI 0.58-0.99 and HR 0.60, 95%CI 0.40-0.89 respectively).

Appendix A: Rates of IID by NS-SEC and explanatory variables

|  |  | **Cases** | **PY**^¥^ | **Rate** | **(95% CI)** |
| --- | --- | --- | --- | --- | --- |
| **NS-SEC** | Managerial/professional | 555 | 2357.9 | 235.4 | (216.6-255.8) |
|  | Intermediate | 161 | 660.0 | 243.9 | (209.0-284.7) |
|  | Routine/manual | 130 | 781.9 | 166.3 | (140.0-197.4) |
|  | Not classifiable | 152 | 783.6 | 194.0 | (165.5-227.4) |
| **Age Group** | <18 | 237 | 678.7 | 349.2 | (307.5-396.6) |
|  | 18-64 | 537 | 2534.6 | 211.9 | (194.7-230.6) |
|  | 65+ | 224 | 1370.2 | 163.5 | (143.4-186.4) |
| **Sex** | Female | 654 | 2759.4 | 237.0 | (219.5-255.9) |
|  | Male | 344 | 1824.0 | 188.6 | (169.7-209.6) |
| **Ethnicity** | White | 979 | 4474.8 | 218.8 | (205.5-232.9) |
|  | Non-White | 19 | 108.7 | 174.8 | (111.5-274.0) |
| **Rurality** | Urban | 688 | 3310.7 | 207.8 | (192.8-223.9) |
|  | Rural | 310 | 1271.0 | 243.9 | (218.2-272.6) |
| **Follow-up** | Email | 663 | 2781.6 | 238.4 | (220.9-257.2) |
|  | Postcard | 335 | 1801.9 | 185.9 | (167.0-206.9) |
| **Employment status** | Employed | 690 | 2857.8 | 241.4 | (224.1-260.1) |
|  | Not working | 301 | 1705.6 | 176.5 | (157.6-197.6) |

^¥^Person-Years

NS-SEC: National Statistics- Socioeconomic Classification; CI: confidence interval

Missing data: Employment status was missing for 30 individuals. Rural-urban classification was missing for three individuals.

**Appendix B: Sensitivity analysis – multiple spells of follow up (n subjects=9332; n failures=1010**)

| **Variable** | **Category** | **Unadjusted** | | **Adjusted^a^** | | **p value** |
| --- | --- | --- | --- | --- | --- | --- |
|  |  | **Hazard ratio** | **(95% CI)** | **Hazard ratio** | **(95% CI)** |  |
| **NS-SEC** | Managerial/professional | 1.0 (reference) |  | 1.0 (reference) |  |  |
|  | Intermediate | 1.04 | (0.88-1.22) | 1.03 | (0.86-1.22) | 0.76 |
|  | Routine/manual | 0.71 | (0.60-0.85) | **0.74** | **(0.61-0.90)** | **0.002** |
| **Rurality** | Urban | 1.0 (reference) |  | 1.0 (reference) |  |  |
|  | Rural | 1.12 | (0.98-1.28) | 1.08 | (0.94-1.25) | 0.28 |
| **Employment status** | Employed | 1.0 (reference) |  | 1.0 (reference) |  |  |
|  | Not working | 0.79 | (0.69-0.91) | 1.00 | (0.83-1.21) | 0.98 |

NS-SEC: National Statistics- Socioeconomic Classification; CI: confidence interval

^a^ Adjusted for all other covariates in the model

Baseline hazard stratified by age group and sex

Missing data: NS-SEC was not classifiable for 1,112 individuals. Employment status was missing for 12 records. Rural-urban classification was missing for four records.

**Appendix C: Sensitivity analysis – all cases (including possible cases) (n subjects=5716; n failures=1152**)

| **Variable** | **Category** | **Unadjusted** | | **Adjusted^a^** | | **p value** |
| --- | --- | --- | --- | --- | --- | --- |
|  |  | **Hazard ratio** | **(95% CI)** | **Hazard ratio** | **(95% CI)** |  |
| **NS-SEC** | Managerial/professional | 1.0 (reference) |  | 1.0 (reference) |  |  |
|  | Intermediate | 1.02 | (0.88-1.19) | 1.02 | (0.88-1.19) | 0.77 |
|  | Routine/manual | 0.74 | (0.63-0.87) | **0.76** | **(0.64-0.89)** | **0.001** |
| **Rurality** | Urban | 1.0 (reference) |  | 1.0 (reference) |  |  |
|  | Rural | 1.07 | (0.94-1.21) | 1.03 | (0.91-1.17) | 0.66 |
| **Employment status** | Employed | 1.0 (reference) |  | 1.0 (reference) |  |  |
|  | Not working | 0.71 | (0.62-0.81) | 0.91 | (0.76-1.07) | 0.26 |

NS-SEC: National Statistics- Socioeconomic Classification; CI: confidence interval

^a^ Adjusted for all other covariates in the model

Baseline hazard stratified by age group and sex

Missing data: Employment status was missing for five individuals. Rural-urban classification was missing for three individuals.

**Appendix D: Sensitivity analysis – Including individuals who were not classifiable by NS-SEC (n subjects=6803; n failures=1355**)

| **Variable** | **Category** | **Unadjusted** | | **Adjusted^a^** | | **p value** |
| --- | --- | --- | --- | --- | --- | --- |
|  |  | **Hazard ratio** | **(95% CI)** | **Hazard ratio** | **(95% CI)** |  |
| **NS-SEC** | Managerial/professional | 1.0 (reference) |  | 1.0 (reference) |  |  |
|  | Intermediate | 1.02 | (0.88-1.19) | 1.03 | (0.88-1.19) | 0.75 |
|  | Routine/manual | 0.74 | (0.63-0.87) | **0.76** | **(0.65-0.89)** | **0.001** |
|  | Not classifiable* | 0.83 | (0.71-0.97) | 0.90 | (0.77-1.06) | 0.20 |
| **Rurality** | Urban | 1.0 (reference) |  | 1.0 (reference) |  |  |
|  | Rural | 1.08 | (0.96-1.21) | 1.05 | (0.93 -1.18) | 0.43 |
| **Employment status** | Employed | 1.0 (reference) |  | 1.0 (reference) |  |  |
|  | Not working | 0.69 | (0.62-0.78) | 0.88 | (0.76-1.02) | 0.10 |

NS-SEC: National Statistics- Socioeconomic Classification; CI: confidence interval

^a^ Adjusted for all other covariates in the model

* Not classifiable for other reasons.

Baseline hazard stratified by age group and sex

Missing data: Employment status was missing for 30 individuals. Rural-urban classification was missing for three individuals.

**Appendix E: Sensitivity analysis – Multiple Imputation of NS-SEC not classifiable group (n subjects=6803**)

| **Variable** | **Category** | **Unadjusted** | | **Adjusted^a^** | | **p value** |
| --- | --- | --- | --- | --- | --- | --- |
|  |  | **Hazard ratio** | **(95% CI)** | **Hazard ratio** | **(95% CI)** |  |
| **NS-SEC** | Managerial/professional | 1.0 (reference) |  | 1.0 (reference) |  |  |
|  | Intermediate | 1.02 | (0.86-1.21) | 1.01 | (0.86-1.20) | 0.87 |
|  | Routine/manual | 0.74 | (0.61-0.90) | **0.78** | **(0.64-0.94)** | **0.01** |
| **Rurality** | Urban | 1.0 (reference) |  | 1.0 (reference) |  |  |
|  | Rural | 1.18 | (1.03-1.35) | 1.14 | (1.00-1.31) | 0.05 |
| **Employment status** | Employed | 1.0 (reference) |  | 1.0 (reference) |  |  |
|  | Not working | 0.74 | (0.65-0.85) | 0.94 | (0.79-1.11) | 0.48 |

NS-SEC: National Statistics- Socioeconomic Classification; CI: confidence interval

^a^ Adjusted for all other covariates in the model

Baseline hazard stratified by age group and sex

Missing data: Employment status was missing for 30 individuals. Rural-urban classification was missing for three individuals.

**Appendix F**: Sensitivity analysis – ten-year age groupings (n subjects=5716; n failures=845)

| **Variable** | **Category** | **Unadjusted** | | **Adjusted^a^** | | **p value** |
| --- | --- | --- | --- | --- | --- | --- |
|  |  | **Hazard ratio** | **(95% CI)** | **Hazard ratio** | **(95% CI)** |  |
| **NS-SEC** | Managerial/professional | 1.0 (reference) |  | 1.0 (reference) |  |  |
|  | Intermediate | 1.04 | (0.87-1.23) | 1.03 | (0.86-1.23) | 0.77 |
|  | Routine/manual | 0.71 | (0.58-0.86) | **0.73** | **(0.60-0.89)** | **0.001** |
| **Rurality** | Urban | 1.0 (reference) |  | 1.0 (reference) |  |  |
|  | Rural | 1.17 | (1.01-1.36) | **1.17** | **(1.01 -1.36)** | **0.03** |
| **Employment status** | Employed | 1.0 (reference) |  | 1.0 (reference) |  |  |
|  | Not working | 0.78 | (0.67-0.91) | 1.06 | (0.86-1.30) | 0.61 |

NS-SEC: National Statistics- Socioeconomic Classification; CI: confidence interval

^a^ Adjusted for all other covariates in the model

Baseline hazard stratified by age group and sex

Missing data: Employment status was missing for five individuals. Rural-urban classification was missing for three individuals.

**Appendix F.1: Sensitivity analysis – age stratified <18 years (n subjects=935; n failures=201**)

| **Variable** | **Category** | **Unadjusted** | | **Adjusted^a^** | | **p value** |
| --- | --- | --- | --- | --- | --- | --- |
|  |  | **Hazard ratio** | **(95% CI)** | **Hazard ratio** | **(95% CI)** |  |
| **NS-SEC** | Managerial/professional | 1.0 (reference) |  | 1.0 (reference) |  |  |
|  | Intermediate | 0.99 | (0.67-1.45) | 0.99 | (0.68-1.46) | 0.98 |
|  | Routine/manual | 0.87 | (0.60-1.26) | 0.89 | (0.61-1.29) | 0.54 |
| **Sex** | Male | 1.0 (reference) |  | 1.0 (reference) |  |  |
|  | Female | 0.98 | (0.74-1.29) | 0.97 | (0.74-1.28) | 0.84 |
| **Rurality** | Urban | 1.0 (reference) |  | 1.0 (reference) |  |  |
|  | Rural | 1.20 | (0.89-1.62) | 1.18 | (0.88-1.59) | 0.84 |
| **Employment status** | Employed | 1.0 (reference) |  | 1.0 (reference) |  |  |
|  | Not working | 0.68 | (0.25-1.82) | 0.71 | (0.26-1.91) | 0.50 |

NS-SEC: National Statistics- Socioeconomic Classification; CI: confidence interval

^a^ Adjusted for all other covariates in the model

**Appendix F.2: Sensitivity analysis – age stratified 18-64 years (n subjects=3310; n failures=463**)

| **Variable** | **Category** | **Unadjusted** | | **Adjusted^a^** | | **p value** |
| --- | --- | --- | --- | --- | --- | --- |
|  |  | **Hazard ratio** | **(95% CI)** | **Hazard ratio** | **(95% CI)** |  |
| **NS-SEC** | Managerial/professional | 1.0 (reference) |  | 1.0 (reference) |  |  |
|  | Intermediate | 1.12 | (0.89-1.41) | 1.09 | (0.87-1.38) | 0.45 |
|  | Routine/manual | 0.73 | (0.56-0.95) | **0.76** | **(0.58-0.99)** | **0.04** |
| **Sex** | Male | 1.0 (reference) |  | 1.0 (reference) |  |  |
|  | Female | 1.44 | (1.17-1.77) | **1.42** | **(1.15-1.74)** | **0.001** |
| **Rurality** | Urban | 1.0 (reference) |  | 1.0 (reference) |  |  |
|  | Rural | 1.12 | (0.92-1.37) | 1.09 | (0.90-1.34) | 0.38 |
| **Employment status** | Employed | 1.0 (reference) |  | 1.0 (reference) |  |  |
|  | Not working | 0.94 | (0.74-1.20) | 0.97 | (0.76-1.24) | 0.81 |

NS-SEC: National Statistics- Socioeconomic Classification; CI: confidence interval

^a^ Adjusted for all other covariates in the model

Missing data: Employment status was missing for two individuals. Rural-urban classification was missing for three individuals.

**Appendix F.3: Sensitivity analysis – age stratified 65+ years (n subjects=1471; n failures=181**)

| **Variable** | **Category** | **Unadjusted** | | **Adjusted^a^** | | **p value** |
| --- | --- | --- | --- | --- | --- | --- |
|  |  | **Hazard ratio** | **(95% CI)** | **Hazard ratio** | **(95% CI)** |  |
| **NS-SEC^b^** | Managerial/professional | 1.0 (reference) |  | 1.0 (reference) |  |  |
|  | Intermediate | 0.95 | (0.65-1.39) | 0.92 | (0.63-1.35) | 0.67 |
|  | Routine/manual | 0.58 | (0.39-0.87) | **0.60** | **(0.40-0.89)** | **0.012** |
| **Sex** | Male | 1.0 (reference) |  | 1.0 (reference) |  |  |
|  | Female | 1.44 | (1.07-1.94) | **1.45** | **(1.08-1.96)** | **0.014** |
| **Rurality** | Urban | 1.0 (reference) |  | 1.0 (reference) |  |  |
|  | Rural | 1.22 | (0.89-1.68) | 1.18 | (0.86-1.63) | 0.31 |
| **Employment status** | Employed | 1.0 (reference) |  | 1.0 (reference) |  |  |
|  | Not working | 1.13 | (0.78-1.64) | 1.12 | (0.77-1.63) | 0.56 |

NS-SEC: National Statistics- Socioeconomic Classification; CI: confidence interval

^a^ Adjusted for all other covariates in the model

Missing data: Employment status was missing for three individuals.

**Appendix G: Sensitivity analysis – Index of Multiple Deprivation (IMD) (n subjects=6803; n failures=991**)

| **Variable** | **Category** | **Unadjusted** | | **Adjusted^a^** | | **p value** |
| --- | --- | --- | --- | --- | --- | --- |
|  |  | **Hazard ratio** | **(95% CI)** | **Hazard ratio** | **(95% CI)** |  |
| **IMD Quintile^b^** | 1 (Most deprived) | 0.74 | (0.56-0.99) | 0.76 | (0.60-1.02) | 0.06 |
|  | 2 | 0.85 | (0.67-1.07) | 0.85 | (0.67-1.07) | 0.17 |
|  | 3 | 0.98 | (0.83-1.17) | 0.98 | (0.82-1.17) | 0.83 |
|  | 4 | 0.93 | (0.78-1.09) | 0.89 | (0.75-1.05) | 0.17 |
|  | 5 (Least deprived) | 1.0 (reference) |  | 1.0 (reference) |  |  |
| **Rurality** | Urban | 1.0 (reference) |  | 1.0 (reference) |  |  |
|  | Rural | 1.18 | (1.03-1.35) | 1.15 | (0.99-1.32) | 0.06 |
| **Employment status** | Employed | 1.0 (reference) |  | 1.0 (reference) |  |  |
|  | Not working | 0.74 | (0.65-0.85) | 0.94 | (0.80-1.12) | 0.50 |

NS-SEC: National Statistics- Socioeconomic Classification; CI: confidence interval

^a^ Adjusted for all other covariates in the model

Baseline hazard stratified by age group and sex

Missing data: Employment status was missing for 30 individuals. Rural-urban classification was missing for three individuals.

**Appendix H: Log-log plot and Kaplan-Meier survival curve for length of time after joining the cohort until occurrence of first episode of IID**

**
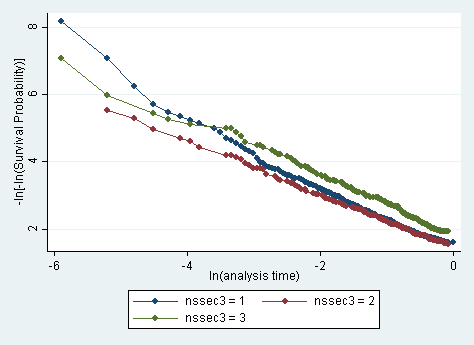
**


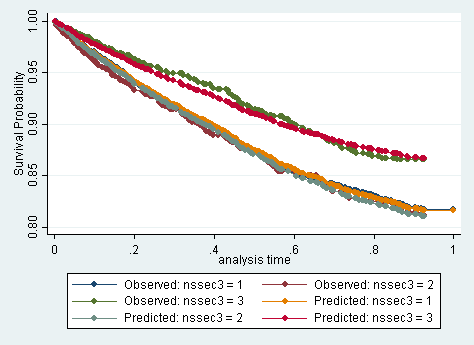

Supplement: Supplementary Data [file ejph-2016-11-om-0896-file003_ckx091.doc]
